# Role of Diabetes in the Prognosis and Therapeutic Outcome of Tuberculosis

**DOI:** 10.1155/2012/645362

**Published:** 2012-04-18

**Authors:** Syed Azhar Syed Suleiman, Daud M. Ishaq Aweis, Ali Jimale Mohamed, Abdul RazakMuttalif, Mohamed A. A. Moussa

**Affiliations:** ^1^School of Pharmaceutical Sciences, USM, 11800 Penang, Malaysia; ^2^Department of Clinical Pharmacy, School of Pharmaceutical Sciences, USM, 11800 Penang, Malaysia; ^3^Ahmadi Hospital, Kuwait Oil Company, Kuwait; ^4^Department of Pharmacology, School of Pharmaceutical Sciences, USM, 11800 Penang, Malaysia; ^5^Faculty of Medicine, Benadir University, Mogadishu, Somalia; ^6^Respiratory Clinic, Hospital Pulau Pinang (HPP), Malaysia; ^7^Department of Community Medicine and Behavioural Sciences, Faculty of Medicine, Kuwait University, Kuwait

## Abstract

*Background*. Increased susceptibility of diabetic mellitus (DM) patients to infection, including tuberculosis (TB), is well documented. The prevalence of DM in Malaysia is reaching epidemic proportions. In this study, we sought to assess risk factors for TB and the impact of DM on the outcome of TB treatment. *Methods*. TB patients, diabetic patients, and diabetic patients with TB were divided into three groups of 200 subjects each. Data were obtained from patients' medical files at the beginning and end of the study period. Prevalence rates of DM and HIV among TB patients were assessed. Prognosis, TB-related complications, anatomical site of infection, and duration of infection and diabetes were also examined. *Results*. The prevalence rates of HIV and DM amongst TB patients were 7.7 and 30%, respectively. The diabetic TB patient group contained more males (72%) and smokers (45.5%) compared to the nondiabetic group (58.3% and 33.5%, resp.). Approximately 74% of diabetic patients were Mycobacterium sputum positive compared to only 51% of nondiabetic patients. Diabetic patients were also more likely to develop pulmonary TB (87%) compared to nondiabetic TB patients (59%). Diabetic TB patients had a higher mortality rate (7.5%) compared to the TB only and DM only groups (1 and 2%, resp.). The duration of TB symptoms was longer in nondiabetic TB patients compared to diabetic TB patients (4.5 versus 2.6 months, resp.). Diabetes antedated TB by a mean time of 4 years. *Conclusions*. We found a higher number of sputum-smear-positive cases and pulmonary TB cases as well as a greater number of males and higher mortality rate in diabetic patients compared to nondiabetic patients.

## 1. Introduction

Tuberculosis (TB) is an infectious disease caused by the intracellular bacterial pathogen, *Mycobacterium tuberculosis*. Approximately one-third of the world's population is currently infected with TB [1]. Major risk factors for developing a TB infection include immunodeficiency diseases, such as HIV/AIDS, as well as poverty, illiteracy, smoking, diabetes mellitus (DM), and silicosis. It is often thought that diabetic patients have compromised immunity, which renders them more susceptible to bacterial, viral, and fungal infections. It has been postulated that diabetes is the leading medical risk factor for TB in Malaysia, and the coexistence of DM and TB increases the complications and cost of treatment. In this study, we sought to determine the prevalence of DM and other TB risk factors as well as the impact of diabetes on the outcome of TB therapy. The results of this study could improve the awareness of clinicians for DM patients infected with TB as well as the treatment approaches used.

## 2. Methods

This was a prevalence-based cohort study evaluating TB treatment outcomes as well as complications of diabetic patients infected with TB. The study was performed at three teaching hospitals: Hospital Pulau Pinang (HPP); Hospital Universiti Sains Malaysia (HUSM) in Kelantan; Universiti Malaya Medical Center in Kuala Lumpur (UM MC, KL). Diabetic patients were primarily selected from HUSM and UMMC; however, since the number of patients from these institutions was not sufficient, some TB cases were also selected from HPP. Patients with severe comorbidities that could affect TB treatment outcome, such as HIV, end-stage renal failure, immunosuppression due to organ transplantation, cancer, systemic lupus erythematosus (SLE), and advanced heart disease were excluded from the outcome analysis.

The study was approved by the Clinical Research Centre of Penang Hospital ((6) dim.SL/CRC/HPP/05), the ethical committee of the Malaya Medical Centre (S13/05/12-2005), and the manager of the Hospital Universiti Sains Malaysia (HUSM/11/020). Patients were grouped into those with TB infection only, with DM only, and with both DM and TB (*n* = 200 patients/group). We evaluated patients with the coexistence of DM and TB, and the remaining groups were used as study controls. We performed a cross-sectional study and prospectively followed patients for at least two years. Each patient's medical file was reviewed at the beginning and at the end of the study (years 2005 and 2008, resp.). Diagnosis of TB was based on laboratory, radiologic, biopsies, and clinical judgments. The prevalence of DM and HIV as well as substance abuse amongst participants was recorded.

Amongst diabetic patients, the type of diabetes was based on the age at disease onset, levels of insulin and C-peptide, and final diagnosis. Insulin was the only pharmacological agent administered to type 1 patients, while type 2 patients received oral hypoglycemic drugs with or without insulin. Diabetic patients were divided into three age groups using a stratified random sampling method. Demographic data, including the patient's health card number, age, gender, race, smoking status, drug abuse, and alcoholic intake as well as clinical variables, including manner of diagnosis, treatment, mortality, and TB-related variables, were recorded. The ethnicity of the subjects was classified as Malays, Chinese, and Indians and other. TB-related variables included the duration of TB symptoms, manner of TB infection, duration of treatment, follow-up period, sputum smear results, outcome of therapy, and anatomical site of TB infection. The durations of diabetes and tuberculosis were studied. All data was recorded by a single researcher. The data were processed using Statistical Package for Social Sciences (SPSS), version 11.5 (IBM, USA). Statistical significance was achieved when *P* ≤ 0.05. The chi-square test was performed to assess association between categorical variables. The Fisher's exact test was used for variables with 2 × 2 contingency tables, and the Pearson's chi-squared test (*x* ^2^) was used for data with larger contingency tables. Normality of quantitative variables was tested. Two tailed *t*-tests and Mann Whitney *U* tests were used to compare normally distributed and nonnormally distributed data sets, respectively. Binary logistic regression was used to evaluate mortality predictors.

## 3. Results

All medical files of diabetic patients below the age of 30 were reviewed because of the limited sample size of this group. Amongst 86 cases, 50 patients were excluded (43 miscoded, 2 cancer patients, and 5 transferred to other centers). Amongst patients between the age of 30 and 59, every 21st patient was selected and 116 patients' files were reviewed. From these files, 33 cases were excluded (2 cancer patients, two SLE patients, and 29 subjects due to lack of information). Among the elder group (60 and above), 96 subjects were chosen by selecting every 13th subject. Fifteen subjects were excluded (13 miscoded, 1 cancer patient, and 1 transferred to another hospital). The three age groups (<30, 30–59, and ≥60 y) contained 36, 83, and 81 subjects, respectively (*n* = 200 patients).

 A total of 1100 medical files of TB-infected patients were reviewed; 291 were misdiagnosed, 41 patients were HIV positive, and 178 subjects were excluded due to other criteria. Two hundred diabetic TB patients were identified, and 200 nondiabetic TB patients were randomly selected from the remaining 390 patients (Table 1). We observed a higher number of Chinese and Indians with both DM and TB than TB alone, while the number of Malays among individuals only infected with TB was higher than the other ethnic groups (64%). Interestingly, the nondiabetic patients infected with TB were younger and weighed less than the other groups (Table 2). The prevalence of smoking was significantly higher in DM patients infected with TB compared to non-DM patients infected with TB (44.5 versus 33.5%, resp.; *P* < 0.01).

We observed a greater number of male smokers than females; amongst smokers, the Chinese ethnic group was the most prevalent (48.8%), followed by Indians (40.8%) and Malays (32.9%). Furthermore, 30.6% of Indians consumed alcohol, compared to 18.6% and 1.4% of Chinese and Malays, respectively (Table 3).

 The prevalence of HIV amongst our subjects was 7.7% (41 HIV+ cases out of 530 TB patients), and the prevalence of DM in TB patients was similar among all centers (~30%). DM antedated TB by a mean time of four years. We also sought to determine the role of DM in exacerbating the duration of TB symptoms, acid-fast bacillus (AFB) sputum smear results, route and site of TB infection as well as TB treatment outcome and related complications. The duration of TB infection was shorter in patients with DM, *P* < 0.05 (Table 3). Interestingly, pulmonary involvement was higher amongst Chinese and Indians compared to Malays (*P* < 0.05). Males also showed greater pulmonary involvement than females, *P* < 0.01 (Table 4).

A significantly higher proportion of sputum-positive cases (*P* < 0.001), greater pulmonary involvement (*P* < 0.001), and higher mortality (*P* < 0.001) were observed in infected diabetic subjects compared to nondiabetic patients (Table 5). Binary logistic regression analysis showed that elder patient who had combination of DM and TB had adjusted odds ratio of 12.3 times risk for death (Table 6).

## 4. Discussion

Few studies to date have assessed the role of diabetes in TB patients. Alisjahbana et al. [2] and Des Bordes [3] studied the prevalence of DM among pulmonary TB patients, and Dooley et al. [4] explored the prevalence and treatment outcome of TB-infected diabetic patients. In addition, Leung et al. [5] assessed the incidence of TB amongst the general population, which included DM as a parameter in the analysis.

Although the higher prevalence of Chinese and Indians among DM-TB group compared to Malays could have been a random occurrence, the possibility that diabetic patients of these two ethnic groups are more susceptible to TB infection cannot be ignored. TB was more prevalent in males, both in diabetic and nondiabetic subjects. Historically, a higher number of males have been found among TB patients compared to females [6]. In our study, nondiabetic patients infected with TB were younger than diabetic patients, which was in agreement with another study [3] and may be attributable to the fact that, while TB typically affects a younger population, DM affects older individuals. We found that infected nondiabetic patients weighed less than infected diabetic patients. Weight and appetite loss are well-documented symptoms in TB patients. However, the difference between diabetic and nondiabetic patients may be due to the fact that the TB pretreatment period was shorter for diabetic patients or the observation that diabetic patients tend to be overweight.

The number of smokers amongst TB patients was nearly double that reported for the general population in the Malaysian Third National Health and Morbidity Survey (NHMS III) in 2006 [7]. Historically, smoking has been considered to be a major TB risk factor, and this hypothesis has been confirmed by recent studies [8, 9]. Although the mechanism has not been established, smoking may compromise immunity and respiratory cilia function [1]. A greater number of males were smokers in our study, which was in agreement with other reports [7, 10, 11]. Chinese patients were more likely to be smokers than Indians or Malaysians. Surprisingly, this finding contradicts the results of Yong et al. [11] and the Malaysian NHMS III [7], which both found that the prevalence of smokers among Malays was greater than Chinese and Indians. This discrepancy may be related to the data recording system in various hospitals as well as the methods applied. It is also possible that Chinese and Indian smokers are more susceptible to TB infection, though this remains to be proven.

The prevalence of alcohol was nearly same for diabetic and nondiabetic infected patients, but was higher compared with the Malaysian general population [7]. Des Bordes [3] also reported a higher prevalence of alcoholism amongst TB-infected patients (49.8%), and other studies have shown that the risk of TB infection increases in persons who consume 40 g of alcohol per day [12]. In addition, a higher prevalence of pulmonary TB and lung cavitations is associated with excess alcohol consumption [13]. In our study, Indians consumed more alcohol than Chinese and Malays, and similar findings were reported by Saroja and Kyaw [14]. The low incidence of alcoholism in Malays may be related to religious beliefs.

We found that the prevalence of DM among TB patients was higher compared to other studies [2, 3, 15] and the general Malaysian population [7]. Historically, reported variations in the prevalence of DM amongst TB patients has varied [6]. In our study, DM antedated TB by a mean time of 4 years. Although DM is considered to be a risk factor for TB, specific precedence time was not reported. The prevalence of HIV amongst TB patients was high compared to less than 0.5% in Malaysian adults between the age of 15–49, according to the UNAIDS 2008 report. This is consistent with other studies, where HIV infection was reported to occur in 8–20% of TB cases [16]. Due to the compromised immune system, there is a far greater likelihood that HIV patients will develop a TB infection.

Greater pulmonary TB involvement was observed in diabetic patients compared to nondiabetic patients, which is consistent with a report by Boucot [6] and Des Bordes [3]. In addition, we observed a higher prevalence of pulmonary TB cases amongst Chinese and Indians, which could be linked to the increased prevalence of smokers, alcoholics, and diabetics in these populations. Greater pulmonary involvement was also observed in males, which was possibly due to socio-demographic factors; males tend to be smokers as well as drug and alcohol abusers compared to females. A greater number of AFB sputum-positive TB cases were also seen in diabetics compared to nondiabetics, and this finding is in agreement with historical [6] as well as more recent studies [3]. The initial duration of infection, which is defined as the time from initial presentation of TB symptoms to commencement of the anti-TB regimen, was higher in nondiabetic infected patients compared to diabetic patients. Verver et al. [17] reported an initial symptom period of two months for actively screened TB patients compared to 2.4 months for patients who passively reported the symptoms. A shorter symptom period in the diabetic patients may be related to greater pulmonary involvement seen in this group, and as such, patients may develop severe symptoms earlier that are easily detectable.

TB cases were divided into new or recurrent infections: recurrence due to relapse (reactivation) is a condition wherein a patient develops TB from bacteria harbored from a previous infection that were not eliminated by antibiotics. Recurrence due to reinfection is due to infection by different bacteria. Although this differentiation is difficult, a case may be considered a relapse case if it occurs within 12 months after completion of the antibiotic regimen and reinfection if TB develops after 12 months. In the current study, a greater number of diabetic patients were infected for the first time compared to nondiabetic patients. However, recurrence in this study did not concur with previous hypotheses suggesting that infection with TB may protect against subsequent reinfection. A similar finding was reported by Verver et al. [17], and greater TB recurrence among HIV-infected patients has been previously reported [18].

In our study, complications attributed to TB were either a result of adverse reactions due to chemotherapy or other complications stemming from TB infection. Although statistically was not significant, higher percentage of TB-infected diabetic patients developed drug-related adverse reactions such as hepatotoxicity, skin allergy, and visual acuity disturbance that required termination and reinitiation of the chemotherapy. Other complications attributable to TB infection were pneumothorax, asthma, COPD, organ disability, and death. Diabetic TB patients showed higher mortality rates, especially among elderly patients, and 13 out of 15 patients that died were above the age of 60. Higher mortality among diabetic tuberculous patient was also reported by Dooley et al. [4]. Historically, patients with combined DM-TB have had a worse prognosis than TB only patients, especially in the era before effective TB therapeutics were available [6].

## 5. Study Limitations

Patients' information was retrieved from medical records, which are subject to incomplete data.

## 6. Conclusions

The prevalence of HIV and DM in TB patients was 7.7% and 29.9%, respectively. A higher number of Chinese and Indians had diabetes and TB compared to other ethnicities, and more pulmonary TB infections were observed in these populations. In addition, we observed a greater number of pulmonary TB cases and higher number of coincidence cases of TB and diabetes in males. Diabetic TB patients had more sputum-smear-positive cases and pulmonary TB infections as well as longer hospitalization periods and higher mortality rates (especially elderly patients) than patients without TB. Diabetic TB patients also experienced a shorter initial symptom period than nondiabetic patients.

## Figures and Tables

**Table 1:** Patient distribution among study centers.

Study groups	All subjects	Excluded subjects			Selected subjects
		Miscoded	HIV	Others	
TB only	1100	291	41	178	200
DM-TB*					200
DM only	298	85	NA	13	200

*DM-TB patients are selected from TB patients.

**Table 2:** Demographic characteristics of study groups.

	Cases (DM-TB)	Controls	
Variables		TB	DM
Gender	*n* (%)	*n* (%)	*n* (%)
Male	144 (72)	116 (58.3)	91 (45.5)
Female	56 (28)	83 (41.74)	109 (54.5)
*P* value (Fisher's exact test)		P* < 0.01	P** < 0.01
Race	*n* (%)	*n* (%)	*n* (%)
Malaysian	97 (49.2)	124 (62)	160 (80)
Chinese	73 (37.1)	55 (27.5)	26 (13)
Indian + others	27 (13.7)	21 (10.5)	14 (7)
*P* value (Pearson *x* ^2^)		P* < 0.05	P** < 0.01
Age			
Mean (SD)	55.1 (12.4)	44.4 (19.2)	52.16 (18)
*P* value (*t*-test)		P* < 0.01	P** NS
Weight			
Mean (SD)	56.1 (11.56)	49.29 (11.3)	63.3 (14)
*P* value (*t*-test)		P* < 0.01	P** < 0.01

P* compares the difference between DM-TB and TB only; P** compares differences between DM-TB and DM only; SD: standard deviation; NS: not significant.

**Table 3:** Substance abuse and TB-related variables among TB groups.

	Cases (DM-TB)	Control (TB)	
Variables			
Substances abuse	*n* (%)	*n* (%)	*P* value
Smokers	89 (44.5)	67 (33.5)	Pearson *x* ^2^: *P* < 0.01
Alcoholic	20 (10)	22 (11)	Pearson *x* ^2^: *P* > 0.05
Druggists	2 (1)	6 (3)	Pearson *x* ^2^: *P* > 0.05
Sputum results			
Positive	148 (74)	102 (51)	Fisher E. T: *P* < 0.01
Negative	30 (15)	76 (38)	
Unknown	22 (11)	22 (11)	
Way of TB infection			
New	181 (90.5)	164 (82)	Pearson *x* ^2^: *P* < 0.01
Recurrent	19 (9.5)	36 (18)	
TB infection Sites			
Pulmonary TB	174 (87)	119 (59.5)	Fisher E. T: *P* < 0.01
Extra pulmonary	21 (10.5)	50 (25)	
Pulmonary + extra	5 (2.5)	31 (15.5)	
TB complications			
Drug reactions	33 (16.5)	28 (14)	Fisher E. T: *P* > 0.05
Other complications	8 (4)	7 (3.5)	
Symptom period in months			
Median (mean)	2 (2.6)	2 (4.5)	*U* test: *P* < 0.05

**Table 4:** Race and gender and anatomical site of TB infection.

Race and site of TB: *n* (%)	PTB	Extra PTB	PTB and extra PTB
Indian	37 (75.5)	7 (14.3)	5 (10.2)
Chinese	108 (83.7)	16 (12.4)	5 (3.9)
Malay	148 (66.7)	48 (21.6)	26 (11.7)
*P* values (Fisher E. T)	*P* < 0.01		

Gender and site of TB *n* (%)	PTB	Extra PTB	PTB and extra PTB

Male	201 (77.3)	41 (15.8)	18 (6.9)
Female	92 (66.2)	29 (20.9)	18 (12.9)
*P* values (Fisher E. T)	*P* < 0.05		

**Table 5:** TB treatment outcome.

	Cases	Controls	
		TB	DM
Treatment outcome	*n* (%)	*n* (%)	*n* (%)
Success	168 (84)	177 (88.5)	N/A
Failure	4 (2)	3 (1.5)	N/A
Death	15 (7.5)	2 (1)	4 (2)
Unknown	13 (6.5)	18 (9)	N/A
*P* value (Fisher E. T)		P* < 0.01	P** < 0.01

P* compares differences between DM-TB and TB only; P**compares differences between DM-TB and DM only; N/A: not applicable.

**Table 6:** Binary logistic regression analysis for death.

Death	Age	Diabetes comorbid	Race	Sex
*P* value	0.003*	0.016*	0.94	0.98

## Abbreviations

DM: Diabetes mellitus
TB: Tuberculosis
HIV: Human immunodeficiency virus
AIDS: Acquired immunodeficiency syndrome
AFB: Acid fast bacillus
COPD: Chronic obstructive pulmonary diseases.

## References

[1] WHO Cummunicable Disease: Tuberculosis Fact Sheet. http://www.searo.who.int/en/Section10/Section2097/Section2106_10682.htm.

[2] Alisjahbana B (2006). Diabetes mellitus is strongly associated with tuberculosis in Indonesia. *The International Journal of Tuberculosis and Lung Disease*.

[3] Des Bordes J (2008). *Factors associated with diabetes in tuberculosis patients in Harris County, Texas 1995–2004*.

[4] Dooley KE, Tang T, Golub JE, Dorman SE, Cronin W (2009). Impact of diabetes mellitus on treatment outcomes of patients with active tuberculosis. *American Journal of Tropical Medicine and Hygiene*.

[5] Leung CC, Lam TH, Chan WM (2008). Diabetic control and risk of tuberculosis: a cohort study. *American Journal of Epidemiology*.

[6] Boucot KR (1957). Diabetes mellitus and pulmonary tuberculosis. *Journal of Chronic Diseases*.

[7] Institute for Public Health (IPH) The 3rd National Health and Morbidity Survey (NHMS III) 2006.

[8] Pednekar MS, Gupta PC (2007). Prospective study of smoking and tuberculosis in India. *Preventive Medicine*.

[9] Gambhir HS, Kaushik RM, Kaushik R, Sindhwani G (2010). Tobacco smoking-associated risk for tuberculosis: a case-control study. *International Health*.

[10] Nissapatorn V, Kuppusamy I, Sim BLH, Kia Fatt Q, Anuar AK (2006). Pulmonary tuberculosis in a hospital setting: gender differences. *Public Health*.

[11] Yong HH, Borland R, Hammond D (2008). Levels and correlates of awareness of tobacco promotional activities among adult smokers in Malaysia and Thailand: findings from the International Tobacco Control Southeast Asia (ITC-SEA) Survey. *Tobacco Control*.

[12] Lönnroth K, Williams BG, Stadlin S, Jaramillo E, Dye C (2008). Alcohol use as a risk factor for tuberculosis—a systematic review. *BMC Public Health*.

[13] Fiske CT, Hamilton CD, Stout JE (2009). Alcohol use and clinical manifestations of tuberculosis. *Journal of Infection*.

[14] Saroja KI, Kyaw O (1993). Pattern of alcoholism in the General Hospital, Kuala Lumpur.. *Medical Journal of Malaysia*.

[15] Mugusi F, Swai ABM, Alberti KGMM, McLarty DG (1990). Increased prevalence of diabetes mellitus in patients with pulmonary tuberculosis in Tanzania. *Tubercle*.

[16] Gandy M, Zumla A (2002). The resurgence of disease: social and historical perspectives on the “new” tuberculosis. *Social Science and Medicine*.

[17] Verver S, Bwire R, Borgdorff MW (2001). Screening for pulmonary tuberculosis among immigrants: estimated effect on severity of disease and duration of infectiousness. *International Journal of Tuberculosis and Lung Disease*.

[18] Picon PD, Bassanesi SL, Caramori MLA, Ferreira RLT, Jarczewski CA, Vieira PRDB (2007). Risk factors for recurrence of tuberculosis. *Jornal Brasileiro de Pneumologia*.

